# Unexpected DNA Loss Mediated by the DNA Binding Activity of Ribonuclease A

**DOI:** 10.1371/journal.pone.0115008

**Published:** 2014-12-11

**Authors:** Federico Donà, Jonathan Houseley

**Affiliations:** Epigenetics Programme, The Babraham Institute, Cambridge, United Kingdom; Univ. of Edinburgh, United Kingdom

## Abstract

Ribonuclease A (RNase A) is widely used in molecular biology research both for analytical assays and for nucleic acid preparation. The catalytic mechanism of RNase A is well understood and absolutely precludes activity on DNA; however anecdotal reports of DNA degradation by RNase A are not uncommon. Here we describe a mechanism by which RNase A treatment can lead to apparent DNA degradation. This results from the surprising finding that RNase A remains functional in a phenol:chloroform mixture, to our knowledge the only enzyme that survives this highly denaturing solvent environment. Although RNase A does not cleave the DNA backbone it is capable of binding to DNA, forming stable RNase A-DNA complexes that partition to the interphase or organic phase during phenol:chloroform purification. The unexpected survival of the RNase A DNA-binding activity in phenol means that these complexes are not dissolved and a substantial amount of RNase A-bound DNA is permanently removed from the aqueous phase and lost on phase separation. This effect will impact DNA recovery from multiple procedures and is likely to represent a source of sequence bias in genome-wide studies. Our results also indicate that the results of analytical studies performed using RNase A must be considered with care.

## Introduction

RNase A ranks among the best characterized proteins known to man, and is used widely in molecular biology applications requiring efficient and specific RNA degradation. In the 1940s Armour & Co. achieved kilogram-scale purification of RNase A making the enzyme commercially available in significant quantities, and this ready availability made RNase A the substrate of choice in the development of many biochemical techniques, reviewed in [1], [2]. RNase A was the subject of landmark studies in protein chemistry including Anfinsen’s Nobel Prize-winning demonstration that the necessary information for protein folding is contained in the primary sequence [3], and was the first active enzyme prepared by total chemical synthesis [4], [5]. In structural biology RNase A was of no less importance, being the third enzyme structure solved by crystallography and the first solved by NMR, as well as providing the model substrate on which methods for studying protein folding were developed [6]–[9].

RNase A was the first enzyme for which the correct catalytic mechanism was deduced [10]. RNA cleavage by RNase A is a two-step process, reviewed extensively in [11]: firstly the enzyme catalyses the nucleophilic attack of the 2′ oxygen on the phosphate backbone, forming a 2′–3′ cyclic phosphate and cleaving the chain. In the second step, the cyclic phosphate is hydrolysed to yield a 3′-phosphate 2′-hydroxy product. The requirement for the 2′ oxygen in the first step of the mechanism explains the absolute specificity of the enzyme for RNA, a property which is invaluable during DNA purification [12].

Here we demonstrate that despite this wealth of understanding, RNase A can act unpredictably, producing unexpected and misleading results during routine RNA analysis. We noted these effects while studying mouse pericentromeric non-coding RNAs; all mouse centromeres (except Y) are composed of minor satellite repeats flanked by extensive arrays of pericentromeric major satellites, and transcripts emanating from minor [13]–[15] and major [14], [16]–[19] satellites have been reported. Major satellite transcripts appear to be processed by Dicer [14], [20]–[22] and are implicated in centromere function and heterochromatin formation [16], [23], [24]. We find that despite apparent RNase A susceptibility, some major satellite species detected by northern blotting in fact emanate from DNA, and that RNase A has a general ability to remove DNA from samples during purification steps.

## Results

**Figure 1:**
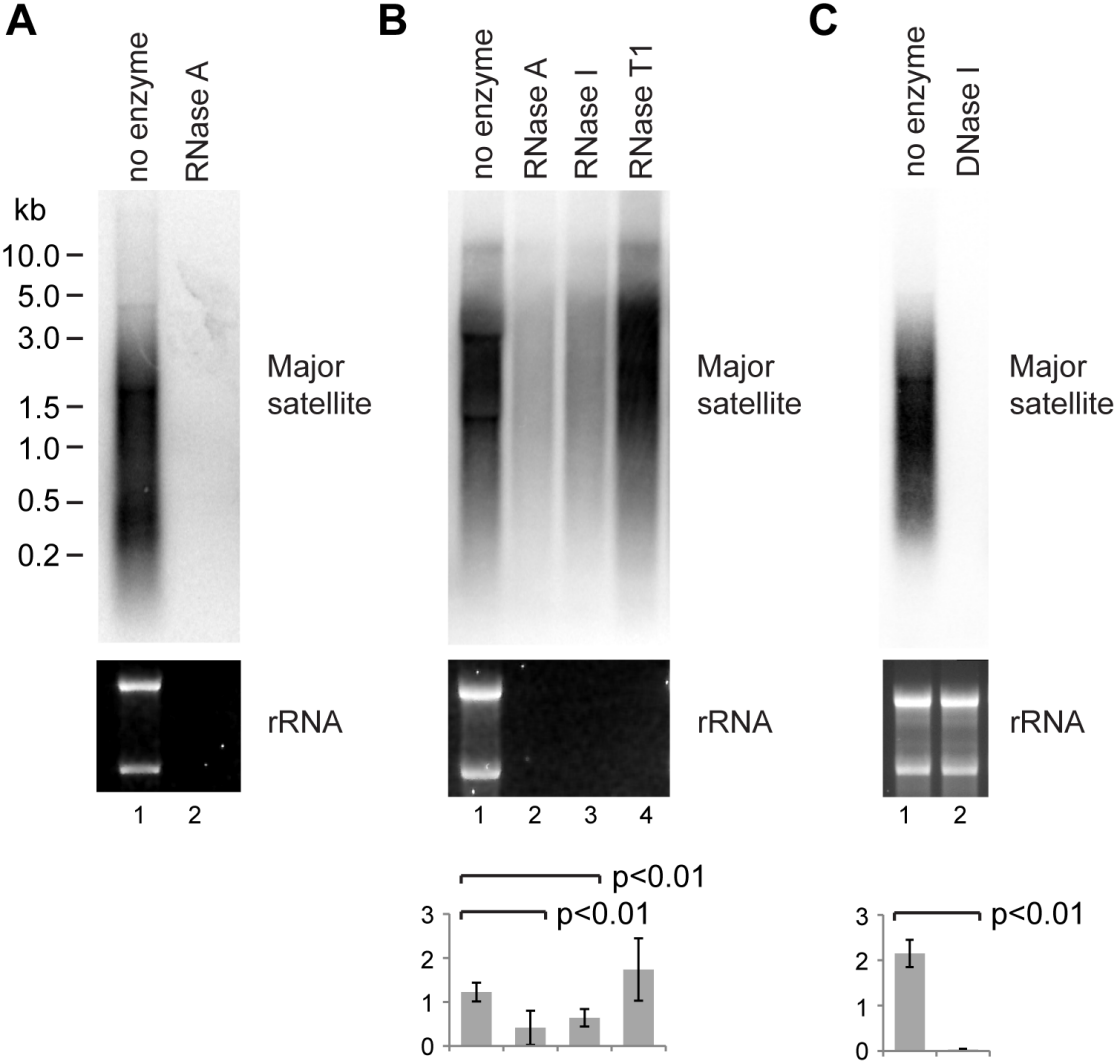
RNase and DNase analysis of major satellite species. Total RNA from NIH/3T3 cells was treated with indicated enzymes for 30 min at 37°, phenol:chloroform extracted, ethanol precipitated and glyoxylated prior to electrophoresis, then blotted and probed for major satellite sequences, ethidium staining of ribosomal RNA is shown as a control. A: Samples treated in water without and with RNase A. B: Samples treated in NEBuffer 3 with indicated ribonucleases. C: Samples treated in RQ1 DNase buffer without and with DNase I. For quantification, error bars represent ±1 s.d., y-axes in arbitrary units, p values calculated using a one-way ANOVA (n = 4) for B and Student’s *t*-test (n = 3) for C.

To better characterize mouse pericentromeric transcripts, we probed northern blots of NIH/3T3 total RNA for major satellite sequences and observed a robust and reproducible signal from species of heterogeneous length (Fig. 1A). To ensure that this signal represented RNA rather than contaminating genomic DNA, we digested samples with RNase A and observed that the signal completely disappeared as expected (Fig. 1A). In Fig. 1A and later figures, samples were incubated without or with RNase A in water for 30 minutes at 37° followed by phenol:chloroform extraction, ethanol precipitation and glyoxylation prior to electrophoresis. NIH/3T3 total RNA analyzed directly on glyoxal gels was indistinguishable from Fig. 1A lane 1.

However, other nuclease digestion experiments yielded confusing results. RNase A or RNase I acting in a mutually compatible buffer significantly reduced the major satellite signal but only by ∼50% (compare Fig. 1A lane 1–2 to Fig. 1B lanes 1–3), the only difference in RNase A treatment conditions between these experiments being buffer composition. Furthermore, RNase T1-treated samples were variable but on average the major satellite signals were not reduced compared to the control despite complete degradation of the ribosomal RNA (Fig. 1B lane 4). These results were not consistent with RNA, and we therefore performed digestions with RNase-free DNase I, resulting in a complete loss of the major satellite signal while the ribosomal RNA remained in-tact (Fig. 1C; equivalent results were obtained using DNase I from another manufacturer – data not shown). These results suggested that the major satellite signals originate from DNA that is aberrantly targeted by RNase A and RNase I.

**Figure 2:**
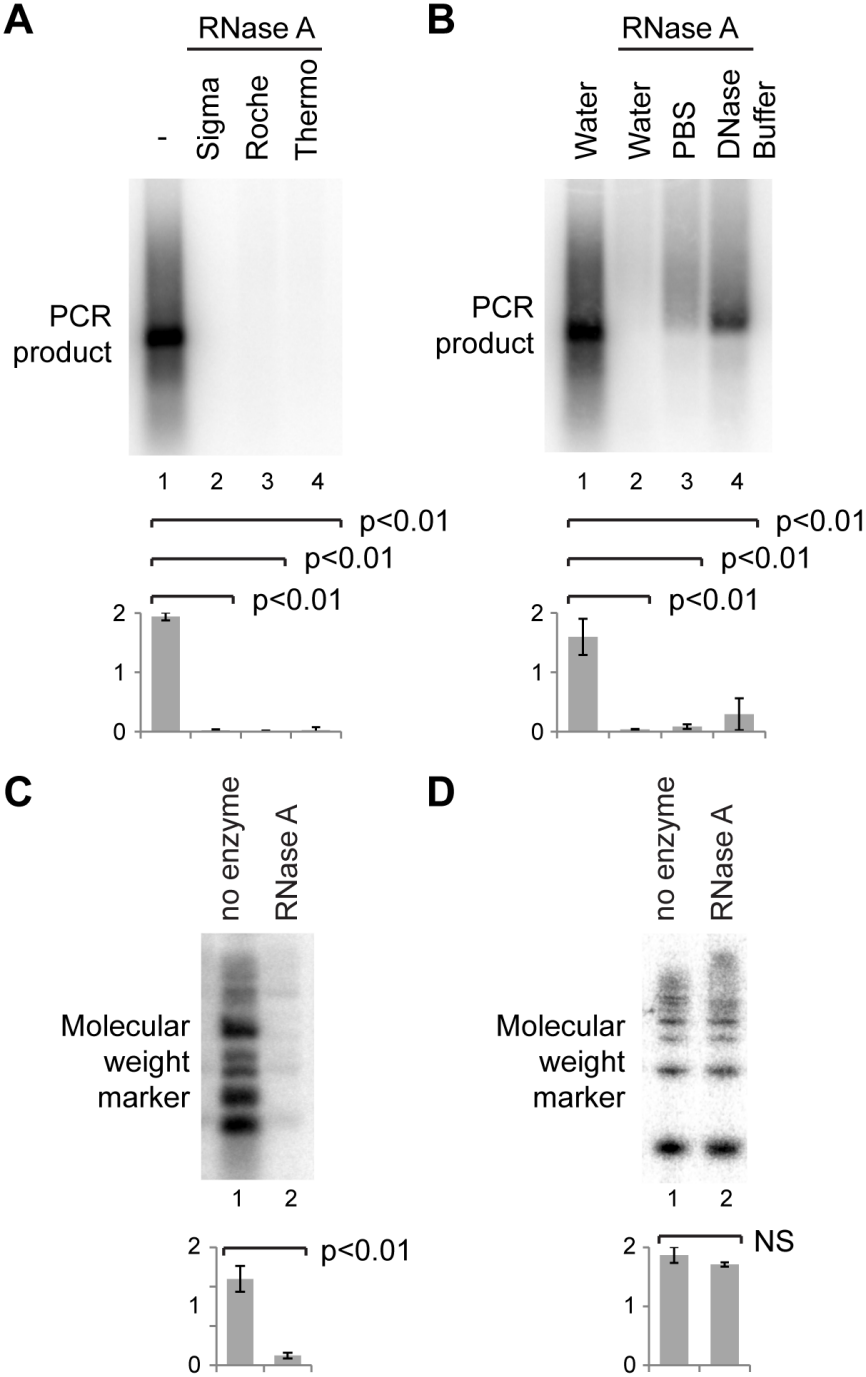
RNase A activity on DNA. A, B: 0.5 ng major satellite PCR product was mixed with 1 µg NIH/3T3 total RNA and analysed as in Fig. 1. A: Treatment with RNase A from different manufacturers. B: Treatment with RNase A in different buffers. C: 50 ng DNA molecular weight marker was mixed with 1 µg NIH/3T3 total RNA and treated without or with RNase A, purified as in Fig. 1 and separated on a non-denaturing 1xTBE gel before blotting and probing for the molecular weight marker. D: ^32^P-labelled 50 bp ladder mixed with 1 µg RNA was treated without or with RNase A for 30 min at 37°, directly separated on an 8% PAGE gel and exposed to a phosphorimaging screen. For quantification, error bars represent ±1 s.d., y-axes in arbitrary units, p values were calculated by one-way ANOVA (A, B) or Student’s *t*-test (C, D), n = 3 in all cases.

To rule out DNase contamination in the RNase A, we prepared RNase A from a different manufacturer exactly as described [12], and also obtained certified DNase-free RNase A from a third manufacturer. All three preparations efficiently removed not only the major satellite signal from total RNA but also a spike of major satellite PCR product (Fig. 2A). This activity was buffer-dependent as PCR-product removal was complete in water but only partial in PBS or DNase I buffer (Fig. 2B). These surprising results clearly demonstrate that RNase A treatment can remove major satellite DNA sequences from RNA samples.

To test the generality of this phenomenon, we asked whether RNase A could also remove DNA molecular weight marker from a sample. Indeed, 50 ng of molecular weight marker mixed with 1 µg total RNA was completely removed on RNase A treatment, showing that the effect is not a peculiarity of major satellite sequences (Fig. 2C). An unreported DNase activity of RNase A seemed unlikely, and we therefore suspected that RNase A treatment instead leads to the loss of DNA during purification, blotting or hybridization. To confirm that DNA is not directly degraded by RNase A in our assays, we treated a radiolabeled low molecular weight DNA marker with RNase A in the presence of RNA, and directly imaged the products after separation through a denaturing polyacrylamide gel (Fig. 2D). As expected, there was no loss of DNA after RNase A treatment in this experiment.

**Figure 3:**
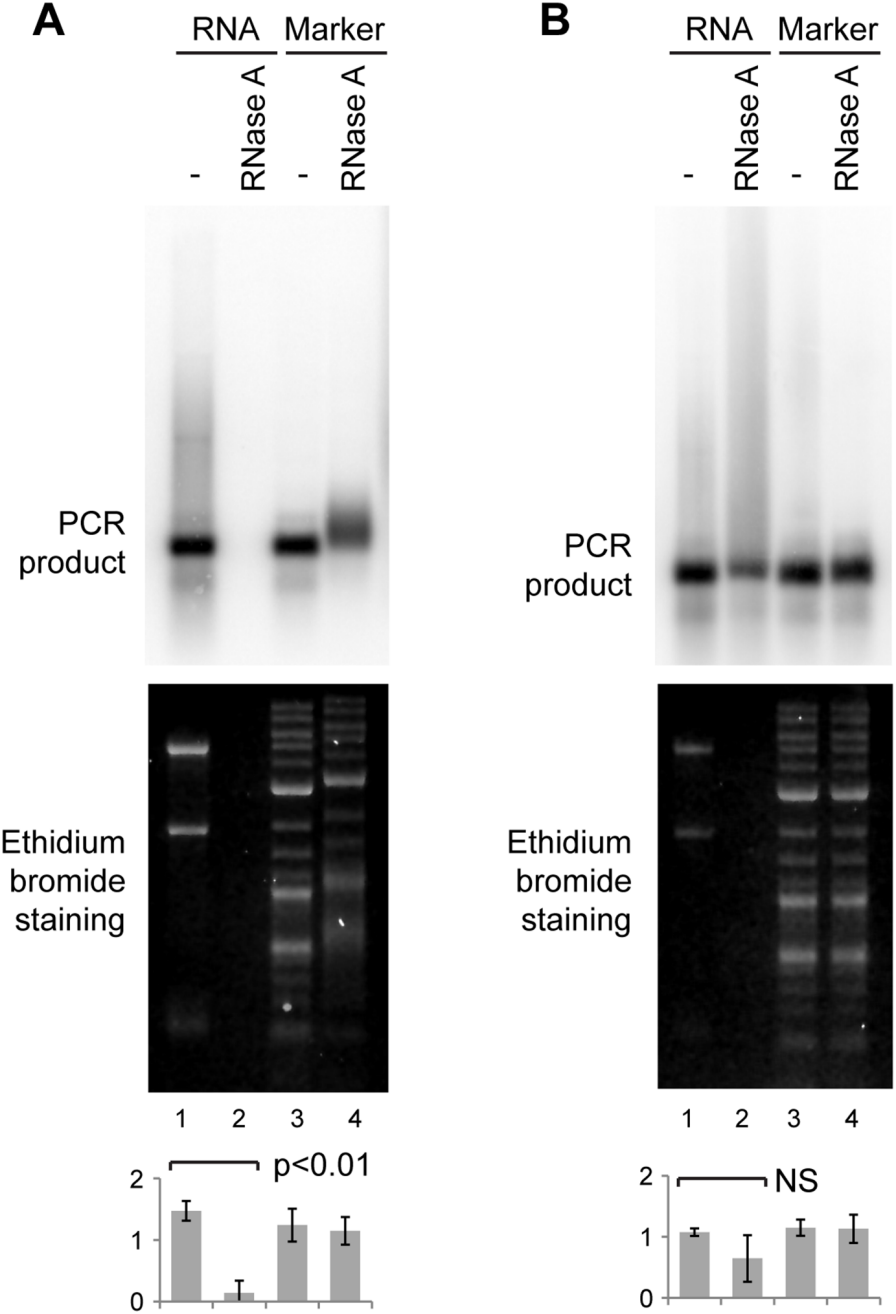
Inhibition of DNA removal by RNase A. A: 0.5 ng major satellite PCR product was mixed with 1 µg NIH/3T3 RNA (lanes 1, 2) or with 1 µg DNA molecular weight marker (lanes 3, 4), and treated without or with RNase A followed by phenol:chloroform extraction and analysis as in Fig. 1. B: Identical to A, except that samples were incubated with 20 µg proteinase K for 15 min at 37° after RNase A treatment and phenol:chloroform extraction was omitted. For quantification, error bars represent ±1 s.d., y-axes in arbitrary units, analysis by one-way ANOVA (n = 3), differences in B are not significant.

RNase A does not degrade DNA but can bind to DNA [25]. If the formation of RNase A-DNA complexes is required for the observed DNA removal, then DNA removal should be inhibited by the presence of excess DNA. Treatment of the PCR product with RNase A in the presence of 1 µg of DNA molecular weight marker prevented loss of major satellite signal (Fig. 3A, quantification). However, migration of the PCR product and also the DNA molecular weight marker was clearly retarded in the RNase A-treated sample compared to the no enzyme control (Fig. 3A lanes 3, 4), suggesting that protein-DNA complexes are indeed formed. Although the binding of RNase A to DNA is known to induce DNA gel migration defects [26], this phenomenon was unexpected here as the reactions in Fig. 3A were purified by phenol:chloroform extraction, ethanol precipitated and then glyoxylated before electrophoresis which would be expected to disrupt any protein-nucleic acid complexes.

The observed change in gel migration suggested that RNase A-DNA complexes survive these highly denaturing treatments. This raised a simple, if surprising, explanation for the removal of DNA by RNase A: if RNase A binds to a DNA molecule at sufficient stoichiometry then the DNA molecule could partition to the interphase or the phenol phase during phenol:chloroform extraction. To test this possibility, we first repeated the assays from Fig. 3A and purified the samples by proteinase K treatment instead of phenol:chloroform extraction. This change abrogated the migration difference in the DNA molecular weight marker and largely prevented the removal of the PCR product when digested in the presence of NIH/3T3 RNA (Fig. 3B). Proteinase K is not particularly active under the RNA-compatible conditions used here (37° for 15 min), and the PCR product digested in the presence of RNA did not return to a clear band but was smeared up towards the wells, suggesting that the removal of RNase A by Proteinase K was only partial (Fig. 3B). Nonetheless, avoiding the phenol:chloroform step clearly reduced the loss of DNA, and the prevention of the band-shift in the molecular weight marker (compare lanes 3, 4 in Fig. 3A, B ethidium panels) shows that this band-shift must be due to RNase A binding as no other protein is present upon which the Proteinase K could act.

**Figure 4:**
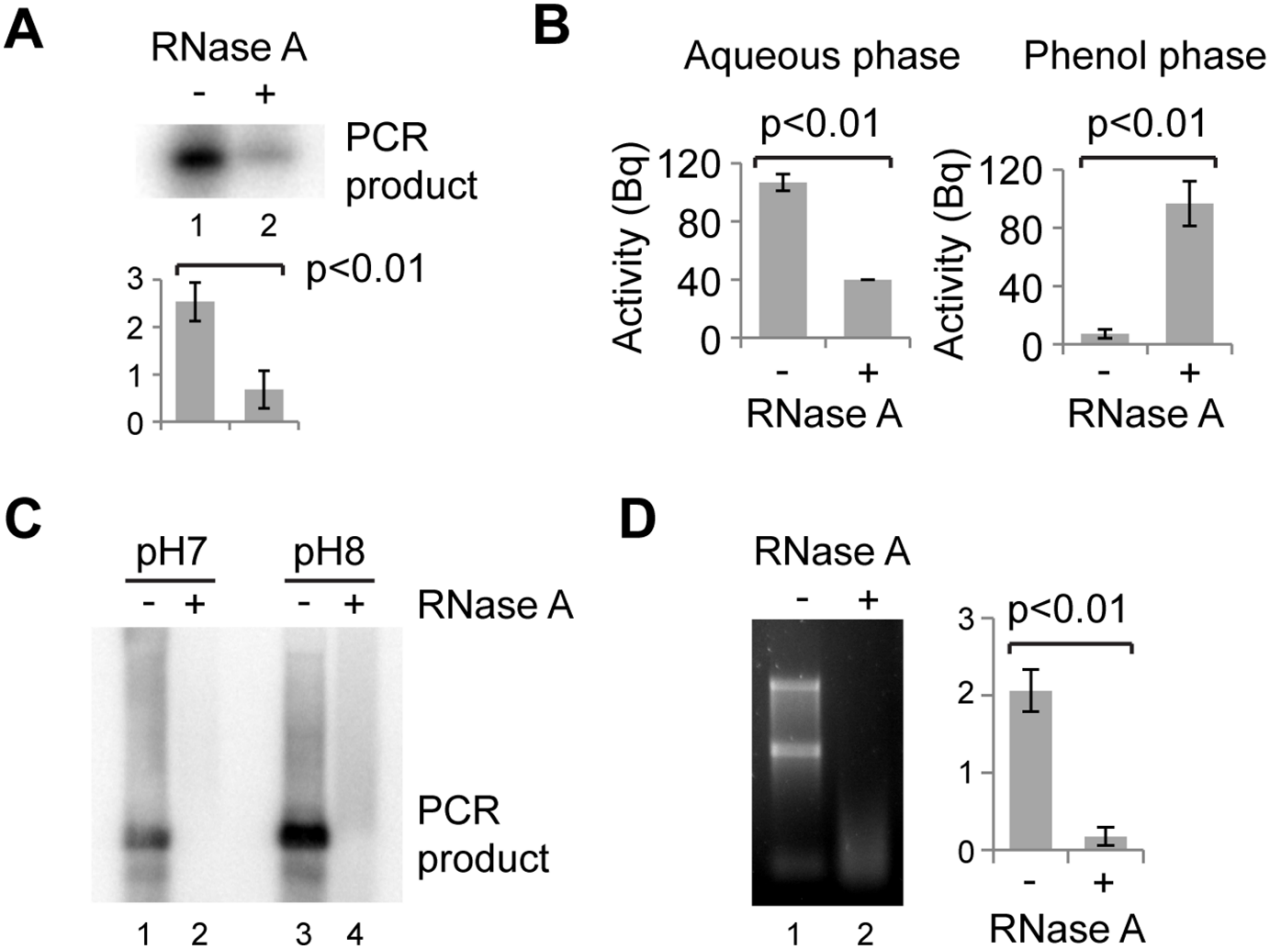
Re-partitioning of RNase-treated DNA. A, B: 5 ng ^32^P-labelled major satellite PCR product mixed with 1 µg of NIH/3T3 RNA was incubated without or with RNase A for 30 min at 37°. Reactions were then diluted to 100 µl with water and extracted with 100 µl phenol:chloroform. A: 10 µl of the aqueous phase was analysed by electrophoresis in a non-denaturing 1xTBE gel which was dried and exposed to a phosphorimaging screen. B: Radioactivity present in the aqueous and phenol phases quantitated using a Geiger counter. C: DNA loss after RNase A treatment with phenol:chloroform extraction at pH 7 or pH 8. D: Activity of RNase A in phenol:chloroform. 1 µl 1 mg/ml RNase A was added to 100 µl phenol:chloroform and vortexed. 1 µl 1 mg/ml NIH/3T3 RNA was added, vortexed and reactions incubated at 37° for 30 min, followed by extraction with 100 µl water, precipitation and gel electrophoresis. For quantification, error bars represent ±1 s.d, y-axes in arbitrary units for A, D or Bequerels in B, analysis by Student’s *t*-test, n = 3.

To directly demonstrate that RNase A can partition DNA to the phenol phase during extraction, we generated radiolabelled major satellite PCR product and treated 5 ng of this with RNase A in the presence of 1 µg total RNA. The reactions were phenol:chloroform extracted and part of the upper phase run directly in a non-denaturing gel, showing that the majority of the radiolabeled PCR product was removed by RNase A treatment (Fig. 4A). The radioactivity of the aqueous and phenol phases was then measured (Fig. 4B) and, consistent with our hypothesis, the radioactivity in the aqueous phase was reduced by RNase A treatment while the radioactivity in the phenol phase dramatically increased. This clearly demonstrates that RNase A can mediate the partitioning of DNA to the interphase or the phenol phase during extraction, explaining the ability of RNase A to remove DNA during extraction.

Partitioning of DNA to the interphase during phenol:chloroform extraction is pH dependent and all these experiments were performed at pH 7, a pH that is appropriate for RNA and small DNA fragments. However, phenol:chloroform extraction for genomic DNA is more routinely performed at pH 8. To ensure that our observations were not simply due to pH, we tested extraction with phenol:chloroform pH 7 and pH 8; complete loss of major satellite PCR product in RNase A treated samples was observed in both cases (Fig. 4C).

The clear retention of the DNA binding activity by RNase A in phenol:chloroform lead us to ask whether the enzyme also remains active. To test this, we added RNase A to phenol:chloroform, vortexed to ensure that the RNase A dissolved into the phenol phase, then added 1 µg RNA. This mixture (RNase A and RNA in 98% water-saturated phenol:chloroform) was incubated at 37° before extraction with water to separate the RNA. Analysis by gel electrophoresis demonstrated that the RNase A successfully degraded the RNA in a reaction mixture almost entirely composed of phenol:chloroform (Fig. 4D).

## Discussion

The DNA binding activity of RNase A has been carefully studied if widely forgotten [25], [27], however our results show that this activity is more tenacious than previously believed. We find that DNA-RNase A complexes are maintained in the presence of 1∶1 phenol:chloroform, generally thought sufficiently denaturing to dissolve any protein-nucleic acid complex. RNase A is exceptionally stable and early purification methods relied on the resistance of the enzyme to boiling or extraction with sulfuric acid [28], [29]; however, this stability does not stem from the maintenance of protein structure under harsh conditions but rather from the ability of the protein to refold back to the active conformation after denaturation. Therefore the survival of the DNA-RNase A complex in phenol:chloroform was unexpected, but not only does the general structure survive we were actually able to observe the activity of RNase A directly in a water saturated phenol:chloroform solution. This demonstrates that although RNase A is solubilized by phenol, it is not denatured.

The resistance of RNase A to phenol:chloroform has untoward consequences in molecular biology applications. Some RNase A-treated DNA molecules are retarded in agarose gels, which alters their apparent molecular weight [26], while others are completely lost though partition to the interphase or phenol phase during phenol:chloroform extraction. Which of these effects is observed in any particular situation will be determined by the stoichiometry of RNase A to DNA; [25] in most experiments presented here the concentration of DNA was low compared to RNase A, allowing many RNase A molecules to bind per DNA molecule and resulting in complexes that act more like proteins and partition to the phenol phase or interphase. However, in Fig. 3A, 2000x more DNA was used (1 µg instead of 0.5 ng), greatly reducing the possible RNase A to DNA ratio. The result suggests that when only a few RNase A molecules bind to DNA, the complexes act more like naked DNA and partition to the aqueous phase allowing recovery of DNA but with RNase A still attached, causing the observed band-shift in Fig. 3A. It is worth noting that this effect is also strongly influenced by the reaction buffer – the binding of DNA by RNase A decreases with increasing salt concentration [25], consistent with our initial observations using RNase A in water or buffer in Fig. 1A, B. Importantly, the quantity of DNA that can be lost during phenol extraction is not small; 1 µg of RNase A efficiently removed 50 ng of DNA molecular weight marker in our hands, and commercial solutions of DNase-free RNase A sold for DNA purification range from 5–40 mg/ml; adding a few microliters of such an enzyme during DNA purification prior to phenol extraction would be predicted to remove upwards of a microgram of DNA depending on the protocol.

Of course, some loss of DNA is often acceptable for downstream applications but only if this loss does not introduce bias into the sample. It is currently unclear whether RNase A could preferentially partition DNA to the phenol phase depending on sequence, but this is likely to be the case. RNase A binds more efficiently to single than double stranded DNA and ‘fixes’ single stranded regions that form through breathing of duplex DNA; this is the basis of the known activity of RNase A as a DNA melting protein [25], [27]. Given that breathing of DNA duplexes is dependent on AT content, it is very likely that RNase A would bind more strongly to AT-rich sequences.

Overall, our data suggests that care is required during routine treatment of DNA samples with RNase A, and that a proteinase K step and/or high salt should always be employed to remove the enzyme prior to phenol chloroform extraction.

## Materials and Methods

NIH/3T3 cells were cultured at 37°C, 5% CO_2_ in DMEM (Sigma) with 10% calf serum. RNA was extracted using 1 ml TRI Reagent (Sigma) per 10 cm^2^ culture dish according to manufacturer’s instructions after washing cells twice with PBS. Major satellite PCR product was amplified using Taq polymerase (NEB) and primers T7 and T3 from pCR4-Maj9-2 [30], digested with *Eco*RI and gel purified using a QIAQuick column (QIAGEN).

RNase A (Sigma R4875) for most experiments was dissolved at 20 mg/ml and boiled for 15 min before dilution to a 1 mg/ml stock solution in Tris pH 7.5 and 50% glycerol. RNase A (Roche 10109142001) was dissolved at 10 mg/ml in 10 mM sodium acetate pH 5.2, boiled for 15 min then neutralised by addition of Tris pH 7.4 to 0.1 M [12] then diluted to a 1 mg/ml stock, 10 mg/ml DNase-free RNase (Thermo EN0531) was used as provided (0.1 µl per reaction). 50 U/µl RNase I_f_ (NEB M0243S) and 1 kU/µl RNase T1 (Thermo EN0541) were used in NEBuffer 3 (Fig. 1C), 1 U/µl DNase I (Thermo EN0521) was used in RQ1 buffer (Promega). 1 µl 20 mg/ml Proteinase K (Roche 3115801) was added to specified reactions.

Enzymatic reactions were performed on 1 µg NIH/3T3 total RNA, 2-log ladder (NEB N3200S) and/or 0.5 ng purified PCR product in 10 µl volumes containing 1 µl enzyme for 30 min at 37°. Reactions were diluted to 100 µl with water and extracted with 100 µl phenol:chloroform pH 7 (Sigma P3803), followed by ethanol precipitation in the presence of 0.3 M NaOAc pH 5.2 and glycogen. Samples were glyoxylated and separated as described on 1.2% gels [12], transferred onto HyBond N+ membrane and probed with random primed major satellite PCR product in UltraHyb Oligo (Life Technologies) at 42°, then washed twice at 42° with 2x SSC 0.5% SDS.

To generate ^32^P- labelled PCR product, major satellite sequences were amplified from pCR4-Maj9-2 as above using Phire polymerase (Thermo) in a normal PCR reaction spiked with 2 µl α-^32^P dCTP and cleaned using a QIAQuick column (QIAGEN). Product fractionation into individual phases was quantified using a hand-held Geiger counter (Mini Series 900 Mini-monitor) by briefly vortexing the sample and placing directly against the detector, counts per second readings were converted to Bq on the assumption that at this distance approximately half the emitted β-particles would be detected.
